# Comparison of continuous wave and cold lateral condensation filling techniques in 3D printed simulated C-shape canals instrumented with Reciproc Blue or Hyflex EDM

**DOI:** 10.1371/journal.pone.0224793

**Published:** 2019-11-21

**Authors:** Anıl Özgün Karatekin, Ali Keleş, Nimet Gençoğlu

**Affiliations:** 1 Department of Endodontics, Faculty of Dentsity, Marmara University, Istanbul, Turkey; 2 Department of Endodontics, Faculty of Dentistry, Ondokuz Mayıs University, Samsun, Turkey; National Taiwan University, school of dentistry, TAIWAN

## Abstract

**Aim:**

To compare the efficiency of continuous wave obturation and cold lateral condensation techniques and filling time in C-shape canals of 3-dimensional (3D)-printed resin teeth shaped with Reciproc Blue (VDW) or Hyflex EDM (Coltene/Whaledent).

**Methodology:**

One tooth with C1-type orifice and root canal morphology and one with C2-type orifice and C2-C3 root canal morphology were selected based on CBCT. Two replicas of selected teeth were manufactured with a 3D-printer and their canals were instrumented with Reciproc Blue or Hyflex EDM. These 4 instrumented replicas were scanned with CBCT. Identical 10 replicas of each group (total of 40) were produced using a 3D-printer and randomly divided into 2 groups (n = 5), root filled with either continuous wave obturation (CW) or cold lateral condensation (LC). Horizontal cross-sections of C1-type were made at 2, 4, 6, 8 mm and C2-type at 2, 4, 6 mm from the apical foramen. Gutta-percha, sealer and void areas were evaluated with image analysis sofware. Data were analysed using nonparametric Kruskal-Wallis and Mann Whitney-U tests and the Factorial ANOVA was used for interaction effects. Time required to fill canals was evaluated using the Mann-Whitney U test.

**Results:**

For C1-type, LC had more gutta-percha and less sealer compared to CW in 2-mm sections (p<0.05). CW had greater percentages of gutta-percha and lower percentages of sealer compared with LC group in 4, 6, 8 mm sections and total area (p<0.05). LC group had higher percentages of voids compared to CW group in 2 and 4 mm sections (p<0.05). For C2-type, CW had more gutta-percha and less sealer versus LC group in all sections and total area (p<0.05). LC had the least gutta-percha and greatest sealer percentages at 6-mm sections (p<0.05). There was no statistically significant difference in the percentages of voids at any level of sections between the filling techniques (p>0.05). In both C-types, there was no significant difference in the percentages of gutta-percha, sealer and voids between Reciproc Blue and Hyflex EDM-shaped groups at any level (p>0.05). Time spent for the LC technique and filling C1-type was significantly longer than when using the CW technique and filling C2-type (p<0.05).

**Conclusions:**

Continuous wave obturation was more effective than lateral condensation in both C1- and C2-type, except for the apical 2 mm section of C1-type, suggesting the need for a modified CW technique.

## Introduction

C shaped root canal morphology is a complex anatomic variation of the root canal system in which two or more canals are connected with mesh or fin-shaped structures as a ‘C’ letter in a continuous form that vary along the cross-sections of a fused root. This configuration was first defined by Cooke&Cox [1].

Though most commonly encountered in mandibular second molars [2], C shaped canal configuration may arise in mandibular premolars and third molars [3–7]. C shaped canal is a rare anatomic diversity with a prevalence greatly varying by geography and ethnicity [8–11]. In Asian population, prevelance of C-shaped canals varies up to 32%-44.6% [11–13]. Although its prevalence is much lower in Turkish population ranging from 4.1% to 8.9% as reported in a number of studies [14–16], the possibility of finding a C shaped root canal anatomy in routine clinical practice should not be underestimated.

Melton et al. [17] suggested a categorization of C shaped canals in 3-types depending on their cross-sectional shape. Subsequently, Fan et al. [18] modified Melton’s way of classification into the following 5-types to provide an evident distinction between them: Type I (C1; continuous “C” shape), Type II (C2; semicolumn morphology), Type III (C3, two or three discrete canals), Type IV (C4, only one round or oval canal in related cross-section), Type V (C5; absence of visible canal lumen). They suggested 3 new standards to identify a canal system as ‘C-shaped’: fused roots; a longitudinal groove on lingual or buccal surface of the root; at least one cross-section of the canal belonging to the C1, C2, or C3 configuration.

C shaped canals cannot not be readily identified through 2-dimensional periapical radiographs and make the cleaning, shaping and obturation procedures challenging without a proper diagnostic method [19–21]. The application of cone-beam computed tomography (CBCT) in endodontic practice may help clinician to effectively diagnose a C-type root canal [18, 19]. These CBCT data may then be used to print 3D replica of a C-type root canal to initiate simulated treatment on 3D printed replica before performing actual treatment on the patient for a better outcome [22, 23].

Single file nickel-titanium (NiTi) instruments with heat-treated special alloys, different manufacturing methods and rotary or reciprocal kinematics have been used increasingly due to their popularity and reduction of instrumentation time and procedural errors in complex canal systems. Unusual form of C shaped canals needs proper shaping and cleaning steps and a convenient obturation technique [24].

A hermetic and void-free obturation is the mainstay of successful root canal therapy. Lateral condensation technique is widely used due to its low cost and more managable apical length control but it requires use of great quantity of additional gutta-percha points specifically in irregular and wide forms like C-configurations. Accessory gutta-perchas does not always form a homogenous mass with the master cone resulting in voids or high percentages of sealer[25, 26]. On the contrary, thermoplasticized gutta-percha obturation techniques are efficient in filling canal irregularities or wide canals which require a high mass of gutta-percha [27–29].

In previous studies, the effect of shaping, cleaning and obturation of C-shaped root canals on extracted human teeth have been examined [30–32]. However, due to possibility of variations between different teeth as well as even in the same tooth and difficulty of reaching an adaquete sample size for pairing teeth with similar root canal anatomy, it was hard to achieve a standardization in these studies. Some other researchers have overcome this limitation either by using a ‘casting method’ [24], which includes several and long steps or ‘3D printing’ [33] but in these experimental designs, only ‘C1’ configuration and one shaping system was used to compare different obturation techniques.

In present study, both C1 and C2-type tooth was included as well as two different novel single file systems with heat treated alloys and different working motions to investigate the quality of root canal fillings obturated with different methods. The aim of this research was to compare the efficiency of continuous wave and cold lateral condensation filling techniques and filling time on C-shape canals of 3D printed resin teeth shaped with Reciproc Blue or Hyflex EDM single file systems.

The null hypothesis tested in the present study was that instrumentation techniques with different kinematics and cross-sectional design would affect the quality of root canal fillings performed by two different filling techniques but there would not be a difference in obturation quality between these 2 different filling methods.

## Materials and methods

### Selection of teeth and CBCT scanning

**Fig 1:**
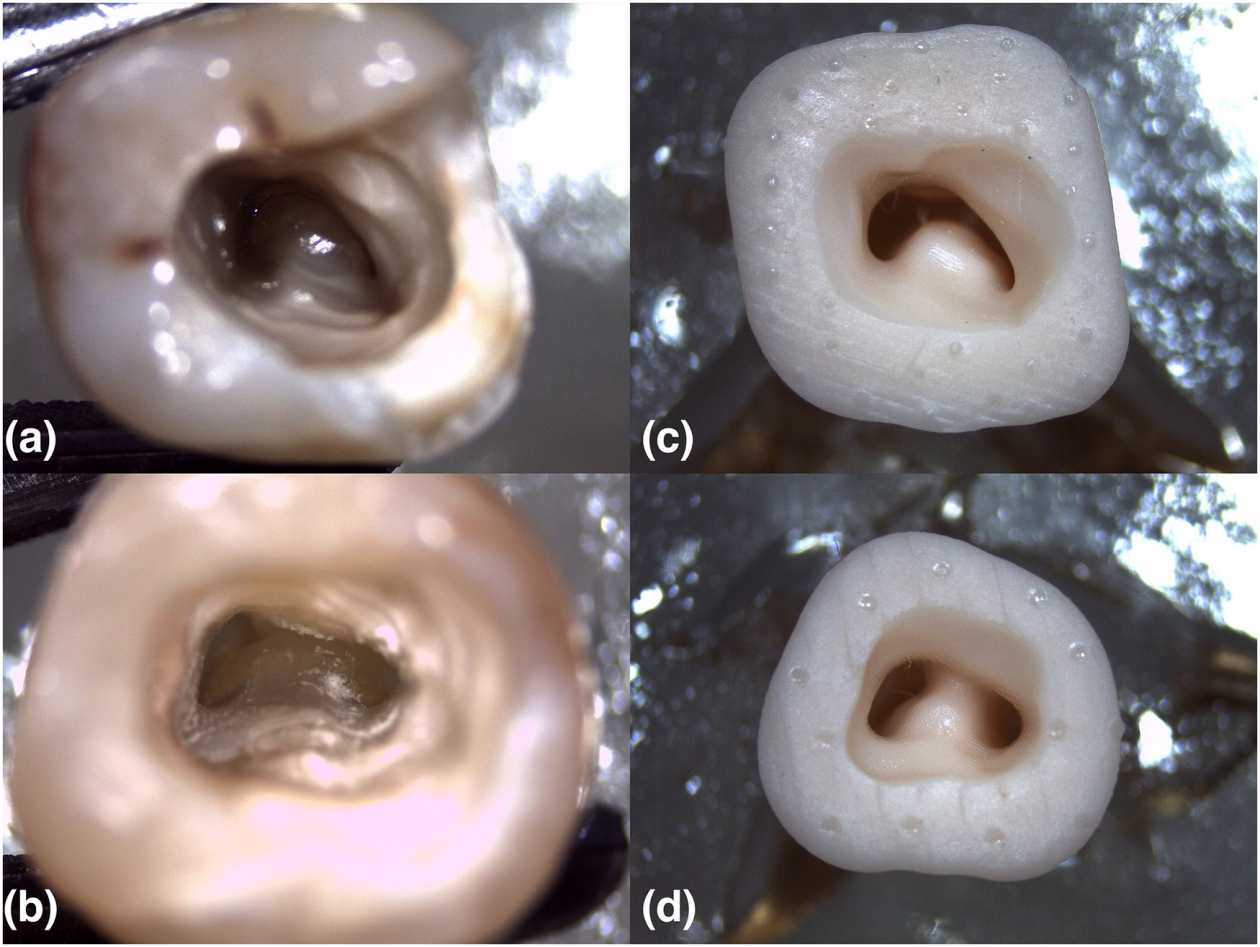
Coronal view of C-types: (A)C1-type mandibular molar, (B)C2-type mandibular molar, (C)C1-type 3D printed resin replica, (D)C2-type 3D printed resin replica.

Extracted human mandibular molars which had fused roots and a longitudinal groove on lingual or buccal surface of the root were sorted out to detect one tooth with C1 root canal morphology and one with C2 according to Fan et al.’s classification [18]. Approval for the use of human teeth was obtained from the Clinical Research Ethics Committee of Marmara University Faculty of Dentistry, Istanbul, Turkey (Approval No. 2018–194), need for consent was waived by the ethics committee. Then, selected teeth were scanned with cone-beam computed tomography (CBCT) (Promax ^®^ 3D Plus; Planmeca, Helsinki, Finland) using the following settings: 75 μm-high resolution endo scanning mode at 90 kV, 8 mA with an exposure time of 12 seconds. Thereafter, cross-sections of teeth obtained from CBCT imaging data were examined with Planmeca Romexis^®^ viewer software and one mandibular molar tooth with C1-type orifice appearance and root canal morphology and one with C2-type orifice appearance and C2-C3 root canal morphology (hereafter referred to as C1 and C2 type) were included in the study (Fig 1A and 1B). For the exact identification of C2-type, α and β angles were calculated with AxioVision Rel. 4.8 software (Zeiss, Göttingen, Germany) on CBCT images at all cross-sections according to Fan et al’s. definition [18]: ‘In C2 type either angle α and β should be not less than 60° and in C3-type there are two or three separate canals and both angles α and β should be less than 60°. Calculations revealed that second tooth with C2-type orifice appearance had a C2-type root canal morphology in the coronal section and C3-type morphology in the middle and apical sections.

### 3D printing of C1 and C2-type mandibular molar replicas for root canal preparation

**Fig 2:**
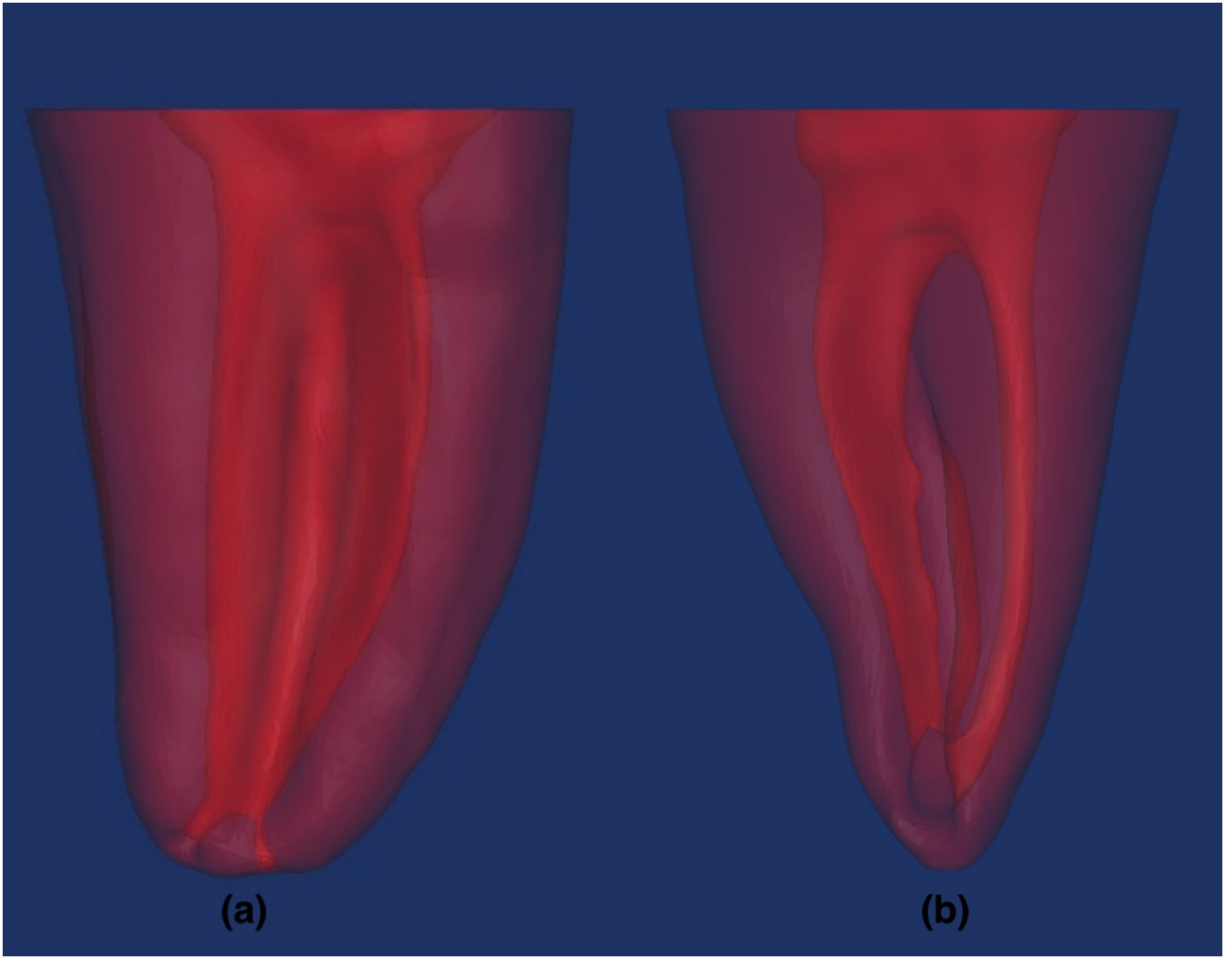
3D reconstructions of (A)C1-type (B)C2-type, lingual views.

CBCT imaging data of the 2 selected teeth in DICOM format were exported to InVesalius 3.0 Software(Centre for Information Technology Renato Archer, Campinas, SP, Brazil) and 3D model of teeth through segmentation was performed; roots of C1 and C2 shape without involving the crowns based on previous studies [33–35] were reconstructed as 12 mm from apex to 2mm above the orifice of canals with Meshmixer^™^ 3.5.474 (Autodesk, San Rafael, CA, USA) and saved in .STL data output format (Fig 2). Then, 3D reconstructed models of teeth were imported to EnvisionTEC Perfactory^®^ Vida HD 3D Printer with advanced DLP technology and 2 replicas of each tooth with C1 and C2-type (4 replicas in total) were manufactured at a resolution of XY:50 and Z:25 μm using EnvisionTec’s E-Model resin printing material (Fig 1C and 1D).

### Root canal preparation of 3D printed C1 and C2-type replicas

Two replicas each for C1-type and C2-type were instrumented with Reciproc Blue and Hyflex EDM single-file systems by the same operator according to the manufacturers’ instructions. During preparation, canals were always irrigated using distilled water and files were used only once to assure the standardization of cutting efficiency. Prior to instrumentation, the working length was determined with 3 different #10 K-files (Mani, Tochigi, Japan) due to the continuous form of canal in C1-type, and in C2-type, the working length was determined with 2 different #10 K-files (Mani, Tochigi, Japan), one file for each of 2 canals. They were passively inserted into canals until the tips were seen from apical foramen and the working lengths were determined 1 mm shorter than the readings obtained at foraminal opening.

First files that could passively reach to the determined working length on both C-types were #10 K-files. So, #10 and #15 21 mm K-files were used for both C shape replicas before the use of reciprocal or rotary instruments in order to establish a safe glide path for both Reciproc Blue and Hyflex EDM files, particularly in the narrow isthmus area of the C1 shape.

#### Instrumentation with Reciproc Blue (R)

Reciproc Blue (R25) file was used with VDW-Gold Reciproc (VDW) endodontic motor in ‘RECIPROC ALL’ mode with in-and-out pecking motion by applying only very light pressure. In C1-type, starting from one of the mesiodistal ends of C shape, instrumentation was carried on until the file reached the specified working length and it was pulled out of the canal and directed to opposite side and these steps were repeated until it reached the other mesiodistal end of canal. In C2-type, instrumentation was carried out for each canal separately in accordance with the manufacturer’s instructions until the file reached the specified working length.

#### Instrumentation with Hyflex EDM (H)

*Hyflex EDM One File* (25/.~) was used with VDW-Gold Reciproc endodontic motor at 400 rpm and 2.5 Ncm torque with tapping movements and without applying any pressure in accordance with the manufacturer’s instructions. In both of C1 and C2 types, rest of the instrumentation procedure was carried out in the same manner of Reciproc Blue instrumentation.

During both operations, continuous irrigation with distilled water and recapitulation with #15 K-file were performed between each insertion of file.

### CBCT scanning of instrumented C1 and C2-type replicas and 3D printing of final replicas

These 4 instrumented resin replicas (C1-Reciproc Blue, C1-Hyflex EDM, C2-Reciproc Blue, C2-Hyflex EDM groups) were then scanned with CBCT (Promax ^®^ 3D Plus; Planmeca, Helsinki, Finland) using the following settings: 75 μm-high resolution endo scanning mode at 90 kV, 8 mA with an exposure time of 12 seconds. Subsequently, aforementioned 3D reconstruction procedure was performed and 10 identical resin final replicas of each group (a total of 40 replicas) were produced with the same 3D printer and 3D printing material that was used in manufacture of initial C-type replicas.

### Filling of final 3D printed C1 and C2-type replicas

Identical final replicas in each group of 4 were randomly divided into 2 groups (n = 5) and were obturated with either continuous wave obturation system (CW) or cold lateral condensation technique (LC) and AH Plus Jet (Dentsply DeTrey, Konstanz, Germany) sealer was used for both techniques. A size 25/.02 paper point covered with sealer was applied on canal walls prior to filling. Furthermore the time spent for filling each 3D-printed resin replica was recorded.

#### Cold lateral condensation technique (LC)

In C1-type, for Reciproc Blue group, one piece of size R25 (Dia-Pro R^™^ Reciproc, Diadent, Chongju, Korea) and for Hyflex EDM group one piece of size 25/~ master gutta-percha (Roeko Hyflex EDM OneFile Guttapercha-Spitzen, Coltene, Langenau, Germany) were coated with sealer and fitted into mesiolingual part of C1-shaped canal. Condensation was performed using size 30 and 25 finger spreaders and 25/.02 and 20/.02 taper accessory gutta-percha cones (Diadent, Chongju, Korea) beginning from the mesiolingual part of the canal to the distolingual part of the canal and then continued around the canal periphery until the spreader could only penetrate 2–3 mm into the canal. Approximately 20–22 accessory gutta- percha cones were used for each replica. The excess length of the cones were cut from the orifice with a #.06–50 heating condenser attached to Duo-Pen (Diadent, Chongju, Korea) and the coronal section of the canals was compacted with a Buchanan Hand Plugger Size 2. In C2-type, filling of the each canal for both of Reciproc Blue and Hyflex EDM instrumentation groups was carried out in the same manner applied to C1-type and approximately 8 accessory gutta-percha cones were used for each replica.

#### Continuous wave obturation system (Dia-Duo, Diadent) (CW)

In C1 type, For Reciproc Blue group, 4 pieces of size R25 (Dia-Pro R^™^ Reciproc, Diadent, Chongju, Korea) and for Hyflex EDM group, 4 pieces of size 25/~ master gutta-percha (Roeko, Hyflex EDM OneFile Guttapercha-Spitzen, Coltene, Langenau, Germany) were covered with sealer and fitted into C1-shaped canal from the mesiolingual part to the distolingual part. Four pieces of tapered gutta-percha cones were needed due to high volume area of the canal shape to maintain stability of the cones and to prevent them moving through isthmus area at the apical part of the canal during down-pack procedure. Then, gutta-percha cones were down packed using continuous wave obturation technique with #.04–50 heating condenser attached to Duo-Pen (Diadent, Chongju, Korea), leaving 3–4mm of the remaining gutta-percha mass in the apical section. Remaining gutta-percha mass was vertically condensed with a Buchanan Hand Plugger Size 1. Then, 2–3 mm of thermoplasticized warm gutta-percha was injected into the canal in 3 stages for backfill process using Duo-Gun (Dia-Duo, Diadent, Chongju, Korea) back fill obturation system with a 23-gauge needle tip. Injected gutta-percha was vertically condensed with a Buchanan Hand Plugger Size 1 in the first step and Buchanan Hand Plugger Size 2 in the second and third steps.

In C2 type, for Reciproc Blue group, 1 piece of size R25 (Dia-Pro R^™^ Reciproc, Diadent, Chongju, Korea) and for Hyflex EDM group, 1 piece of size 25/~ taper master gutta-percha (Roeko, Hyflex EDM OneFile GuttaPercha-Spitzen, Coltene, Langenau, Germany) were covered with sealer and fitted into each of the 2 canals and rest of the procedure was carried out in the same manner applied to C1-type.

One week after filling, sectioning of C-shape replicas was performed using a 0.3 mm-thick diamond blade (IsoMet^™^ 15HC, Buehler, Lake Bluff, IL, USA) with a precision cutter device(IsoMet^™^ 1000, Buehler, Lake Bluff, IL, USA) under water irrigation at 200 rpm speed setting. Horizontal cross-sections of C1 group were made at 2, 4, 6 and 8 mm and for C2 group 2, 4, 6 mm from the apical foramen of the specimens because the apical foraminal exit of the C2-type was 2mm above the apex and the length of replica was not enough for an 8 mm sectioning. The images of cross-sections were taken with a stereomicroscope(Leica EZ4 HD) at 32x magnification, then the areas of gutta-percha, sealer and voids were evaluated with with AxioVision Rel. 4.8 image analysis software (Zeiss, Göttingen, Germany). Then, percentage of gutta-percha, sealer and voids were calculated.

## Statistical analysis

All statistical analyses were performed with SPSS Statistics, Version 25.0 software (IBM, Chicago, IL). Since the normality assumptions were violated, nonparametric Kruskal-Wallis and Mann Whitney-U tests were performed to investigate the percentages of gutta-percha, sealer and voids depending filling techniques, shaping system and the level of sections in C shaped canals (C1 and C2-type). Additionally, after checking the main effects by Mann-Whitney U and Kruskal-Wallis tests, the Factorial ANOVA was used to see the interaction effects of variables on gutta-percha, sealer and voids. The mean time spent for filling of C shape replicas was examined by conducting nonparametric Mann-Whitney U test since the normality assumption was violated. Statistical significance level was set at *p* <0.05.

## Results

### Time required for filling

Time spent for filling C1-type was significantly longer than for C2-Type (p < 0.05). However, there was no statistically significant difference in time spent for filling between *Reciproc Blue* and *Hyflex EDM* systems (*p>0*.*05*). Table 1 shows the descriptive statistics and Mann- Whitney U Test results.

**Table 1:** Descriptive statistics of time spent(in minutes) for filling and the results of Mann-Whitney U group statistics.

	Lateral Condensation				Continuous Wave			
	*n*	*Median*	*IQR*	*p*	*n*	*Median*	*IQR*	*p*
*C-Type*								
C1	10	14.09^a^	3.14	< .001	10	9.72^a^	1.27	< .001
C2	10	9.51^a^	0.91		10	8.37^a^	0.34	
*Shaping System*								
R	10	11.74	4.78	.33	10	8.78	1.20	.94
H	10	10.82	4.75		10	8.48	1.85	

^a^ Statistically significant difference between groups at level of p < .001

For both of C1 and C2-types, the Mann-Whitney U test was conducted to find out the difference between filling techniques by the time spent for filling. In C1-type, the results (Table 1) indicate that lateral condensation technique (*Md* = 14,09, IQR = 3,14) was associated with significantly longer time for filling than the continuous wave technique (*Md* = 9,72, IQR = 1,27) (*p* < 0.05). In C2-type, the results (Table 1) indicate that lateral condensation technique (*Md* = 9,51, IQR = 0,91) was associated with significantly longer time for filling than the continuous wave technique (*Md* = 8,37, IQR = 0,34) (*p* < 0.05).

### Percentages of canal area occupied by gutta-percha, sealer and voids

#### C1-type

A total of 80 sections from 20 C1-type replicas were evaluated and the main effects were analysed by Mann-Whitney U and Kruskal-Wallis tests. The findings indicated that there were significant differences between cold *lateral condensation* and *continuous wave obturation* techniques by percentages of gutta-percha, sealer and voids at each level independent from shaping systems (*p* < 0.05). Continuous wave technique had significantly higher gutta-percha and lower sealer and void percentages than lateral condensation technique on average in all sections. However, there were no statistically significant differences in the percentages of gutta-percha, sealer and voids between *Reciproc Blue* and *Hyflex EDM* systems at any level (*p>0*.*05*). Also, for the percentages of gutta-percha, sealer and voids; the difference between the level of sections (2mm, 4mm, 6mm, 8 mm and total) was significant irrespective of filling techniques (*p*<0.05). 2-mm sections had significantly less gutta-percha and more sealer compared with 4, 6 and 8 mm sections (*p* < 0.05). The highest percentages in gutta-percha and lowest in sealer was at 8-mm level of sections. 4 mm level of sections showed the highest and 2 mm level of section showed the lowest voids percentages. Tables 2, 3 and 4 show all descriptive statistics for the variables and *p* values for all group differences in C1-type.

**Table 2:** Mann-Whitney U and Kruskal-Wallis tests results with descriptive statistics of C1-type’s median percentage values of gutta-percha, sealer and voids according to filling techniques, shaping system and level of sections.

		Gutta-Percha			Sealer			Voids		
	*n*	*Median*	*IQR*	*p*	*Median*	*IQR*	*p*	*Median*	*IQR*	*p*
*Filling Techniques*										
LC	40	86.40^c^	8.85	< .001	13.10^c^	8.94	< .001	0.16^a^	0.34	< .05
CW	40	97.30^c^	5.75		2.50^c^	5.89		0.00^a^	0.13	
*Shaping System*										
R	40	89.66	17.67	.25	10.96	17.70	.30	0.09	0.32	.36
H	40	94.06	12.05		5.54	12.12		0.02	0.21	
*Level of Sections*										
2mm	20	80.40^c^	23.88	< .001	19.26^c^	23.88	< .001	0.00^b^	0.00	< .01
4mm	20	91.13^c^	13.68		8.79^c^	13.40		0.00^b^	0.23	
6mm	20	92.71^c^	11.43		6.93^c^	11.80		0.18^b^	0.30	
8mm	20	97.06^c^	6.80		2.52^c^	6.79		0.12^b^	0.27	
Total	20	91.27^c^	6.51		8.49^c^	6.46		0.15^b^	0.26	

^a^ Statistically significant difference between groups at level of p < .05,

^b^ p < .01,

^c^ p < .001

**Table 3:** Descriptive statistics and Kruskal-Wallis test results on the percentages of gutta-percha, sealer and voids according to level of sections in C1-type.

		Gutta-Percha			Sealer			Voids		
	*n*	*Median*	*IQR*	*p*	*Median*	*IQR*	*p*	*Median*	*IQR*	*p*
*2mm*										
R-LC	5	89.89^a^	7.88	< .05	10.10 ^a^	8.16	< .05	0.00	0.95	.20
H-LC	5	84.13 ^a^	8.96		15.86 ^a^	9.03		0.00	0.61	
R-CW	5	65.44 ^a^	5.55		34.55 ^a^	5.55		0.00	0.00	
H-CW	5	53.57 ^a^	34.08		46.42 ^a^	34.08		0.00	0.00	
*4mm*										
R-LC	5	73.37 ^b^	10.32	< .01	24.49 ^b^	11.67	< .01	0,34 ^a^	1.75	< .05
H-LC	5	85.47 ^b^	5.75		14.37 ^b^	5.76		0,10 ^a^	0.17	
R-CW	5	97.64 ^b^	2.45		2.35 ^b^	2.45		0,00 ^a^	0.00	
H-CW	5	97.18 ^b^	3.30		2.81 ^b^	3.16		0,00 ^a^	0.14	
*6mm*										
R-LC	5	80.39 ^b^	5.48	< .01	19.30 ^b^	5.35	< .01	0,26	0.44	.08
H-LC	5	86.66 ^b^	2.95		13.33 ^b^	2.99		0.00	0.26	
R-CW	5	96.67 ^b^	3.30		2.44 ^b^	3.17		0,28	0.47	
H-CW	5	99.13 ^b^	3.54		0.80 ^b^	3.48		0,06	0.18	
*8mm*										
R-LC	5	89.42 ^b^	5.43	< .01	10.35 ^b^	5.43	< .01	0,00 ^b^	0.11	< .01
H-LC	5	96.18 ^b^	3.14		3.53 ^b^	3.05		0,30 ^b^	0.45	
R-CW	5	99.09 ^b^	2.61		0.63 ^b^	2.78		0,13 ^b^	0.30	
H-CW	5	99.43 ^b^	0.55		0.49 ^b^	0.48		0,04 ^b^	0.09	
*Total*										
R-LC	5	82.25 ^b^	5.97	< .01	17.57 ^b^	6.14	< .01	0,34 ^a^	0.59	< .05
H-LC	5	90.09 ^b^	2.27		9.72 ^b^	2.33		0,18 ^a^	0.20	
R-CW	5	93.78 ^b^	2.83		5.86 ^b^	2.82		0,16 ^a^	0.20	
H-CW	5	95.52 ^b^	5.84		4.43 ^b^	5.85		0,04 ^a^	0.07	

^a^ Statistically significant difference between groups at level of p < .05,

^b^ p < .01

**Table 4:** Descriptive statistics and Kruskal-Wallis test results on the percentages of gutta-percha, sealer and voids according to level of sections independent from shaping system in C1-type.

		Gutta-Percha			Sealer			Voids		
	*n*	*Median*	*IQR*	*p*	*Median*	*IQR*	*p*	*Median*	*IQR*	*p*
*2mm*										
LC	10	85.81^b^	8.82	< .01	13.84^b^	8.72	< .01	0.00^a^	0.67	< .05
CW	10	65.33^b^	15.67		34.66^b^	15.67		0.00 ^a^	0.00	
*4mm*										
LC	10	84.35^c^	12.43	< .001	15.41^c^	13.48	< .001	0.17 ^a^	0.54	< .05
CW	10	97.47^c^	2.33		2.53^c^	2.13		0.00 ^a^	0.00	
*6mm*										
LC	10	86.12^c^	6.95	< .001	13,77^c^	6.92	< .001	0.18	0.31	.76
CW	10	97.25^c^	3.33		2.17^c^	3.35		0.18	0.27	
*8mm*										
LC	10	92.64^b^	7.18	< .01	7.25^b^	7.36	< .01	0.21	0.36	.65
CW	10	99.42^b^	0.58		0.51^b^	0.77		0.1	0.13	
*Total*										
LC	10	87.56^c^	8.01	< .001	12.13^c^	8.04	< .001	0.23 ^a^	0.32	< .05
CW	10	93.88^c^	4.71		5.84^c^	4.72		0.10 ^a^	0.13	

^a^ Statistically significant difference between groups at level of p < .05,

^b^ p < .01,

^c^ p < .001

**Fig 3:**
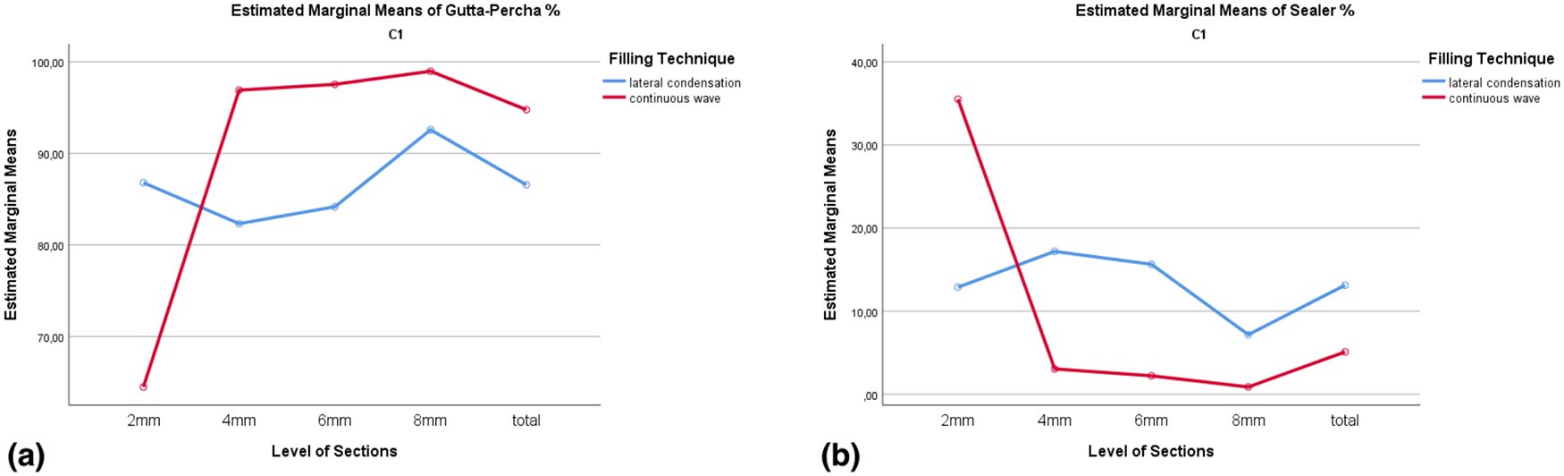
Interaction effects of filling techniques and level of sections on (A)gutta-percha (B)sealer percentages in C1-type.

The factorial ANOVA was conducted to explore interaction effects of filling techniques and level of sections. The interaction effect of those variables was significant for percentages of gutta-percha and sealer (p < 0.05) but not for voids percentages (p > 0.05). The interaction effects could be explained by differences between filling techniques in the ratio of gutta-percha and sealer depending on the sectional levels. Fig 3A and 3B shows the interaction effects in C1-type. In the 2mm level of section, the behavior of the line was different than the others.

**Fig 4:**
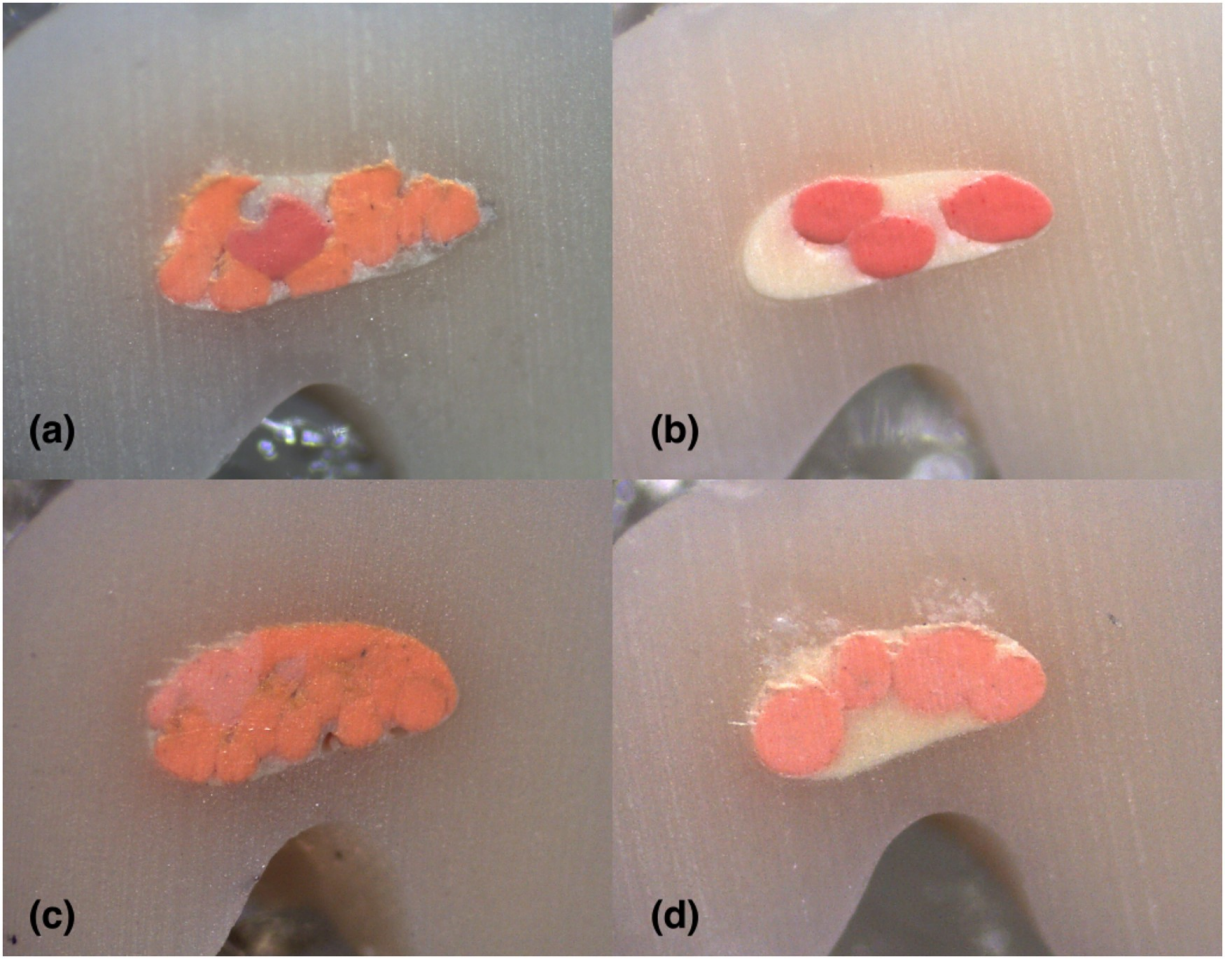
Stereomicroscope images of 2 mm sections instrumented with Hyflex EDM: (A) LC (B) CW; Stereomicroscope images of 2 mm sections instrumented with Reciproc Blue: (C) LC (D) CW in C1-type.

**Fig 5:**
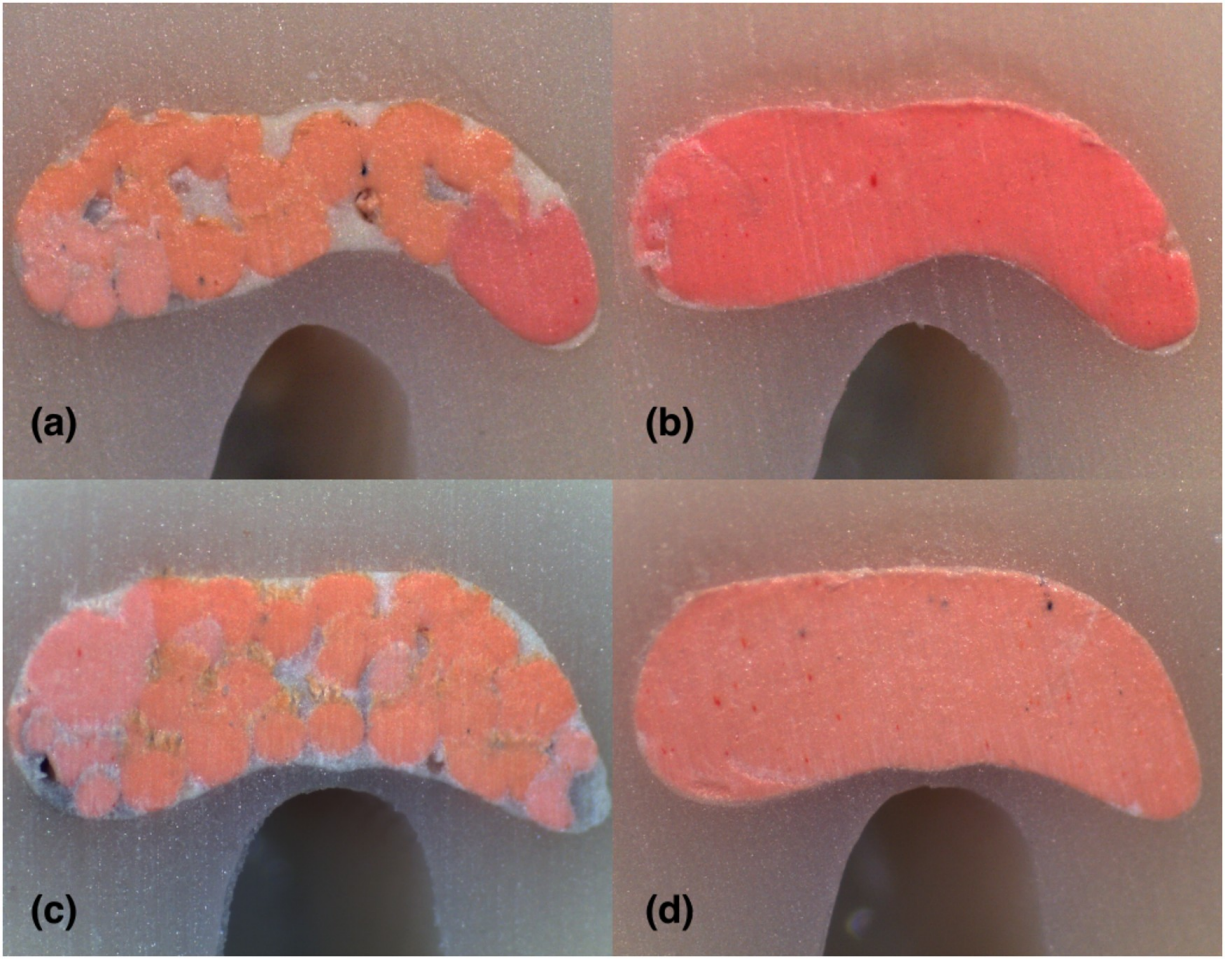
Stereomicroscope images of 4 mm sections instrumented with Hyflex EDM: (A) LC (B) CW; Stereomicroscope images of 4 mm sections instrumented with Reciproc Blue: (C) LC (D) CW showing the resin thickness difference between shaping systems in groove area of C1-type.

**Fig 6:**
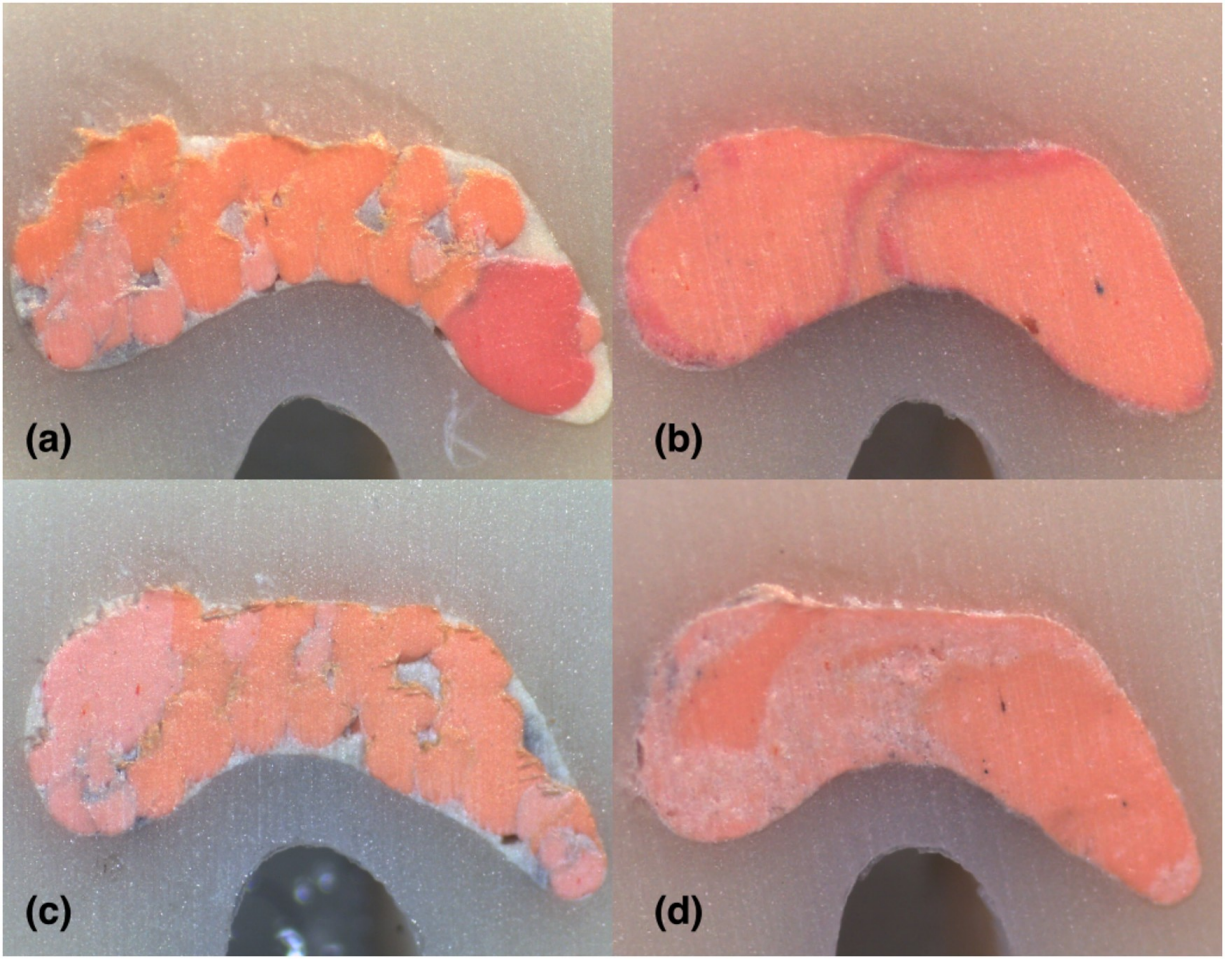
Stereomicroscope images of 6 mm sections instrumented with Hyflex EDM: (A) LC (B) CW; Stereomicroscope images of 6 mm sections instrumented with Reciproc Blue: (C) LC (D) CW in C1-type.

**Fig 7:**
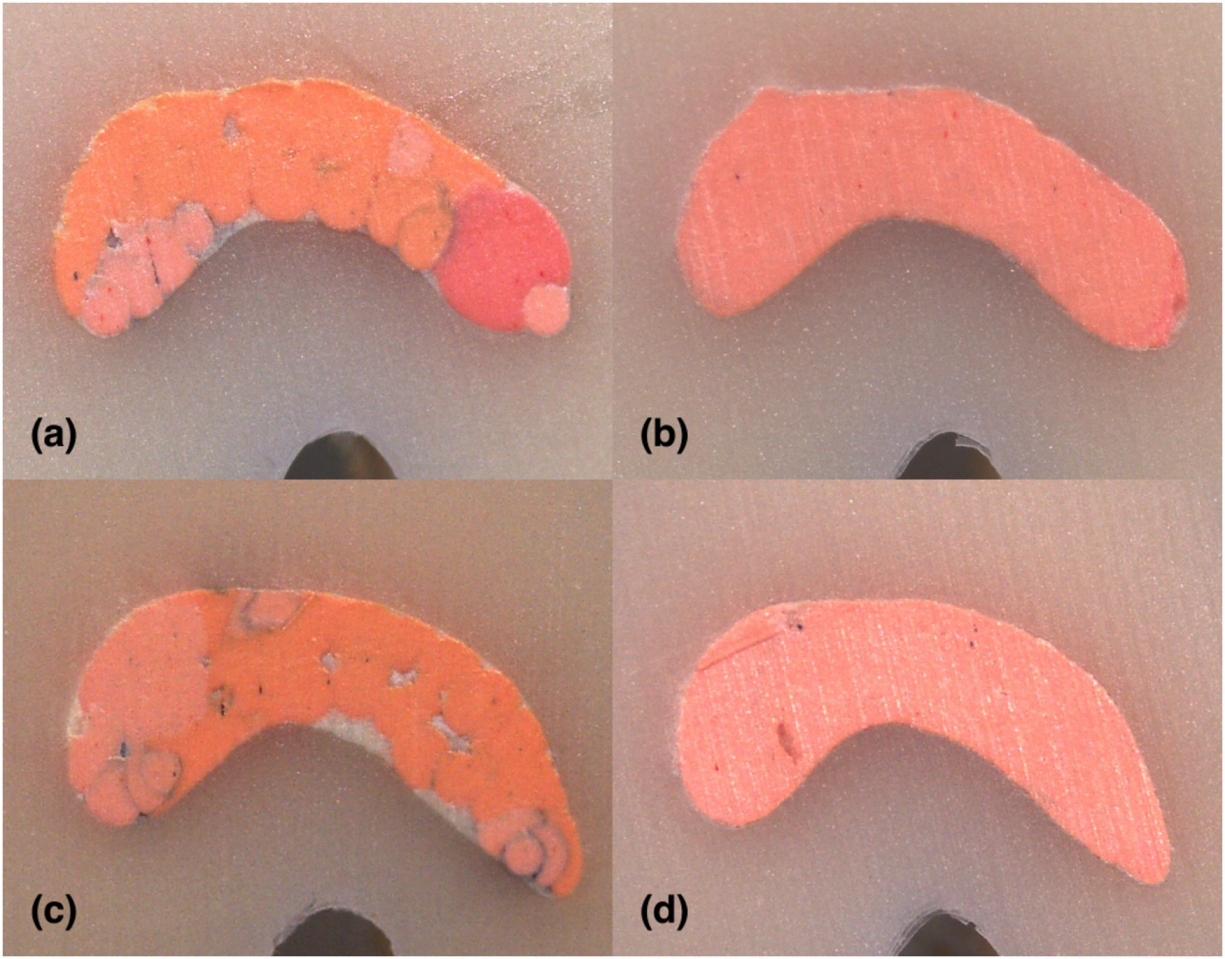
Stereomicroscope images of 8 mm sections instrumented with Hyflex EDM: (A) LC (B) CW; Stereomicroscope images of 8 mm sections instrumented with Reciproc Blue: (C) LC (D) CW in C1-type.

For the investigation of both shaping systems’ and filling techniques’ impact on the percentages of gutta-percha, sealer and voids by controlling the sectional levels, non-parametric Kruskal-Wallis test was conducted. The analysis revealed a statistically significant difference between the filling techniques in the percentages of gutta-percha and sealer for all levels of sections (p<0.05) and statistically significant differences in the percentages of voids for 2mm, 4mm level of sections and total area (p<0.05). LC group had higher gutta-percha and lower sealer percentages compared to CW group in 2-mm sections (P < 0.05) (Fig 4). CW group had more gutta-percha and less sealer compared with LC group in 4, 6, 8 mm sections and in the total area (p<0.05) (Figs 5–7). The mean percentages of voids in LC group were significantly higher compared to CW group in 2 and 4 mm sections (p<0.05). Both LC and CW groups had the highest gutta-percha and lowest sealer percentages in 8 mm sections(Fig 7). CW group had the lowest gutta-percha and highest sealer percentages at 2 mm sections.

#### C2-type

A total of 60 sections from 20 C2-type replicas were evaluated and the main effects were explored by Mann-Whitney U and Kruskal Wallis tests. The findings indicated that there were significant differences between cold *lateral condensation* and *continuous wave* obturation techniques by the percentages of gutta-percha and sealer (*p* < 0.05) but no significant difference was found between the techniques in the percentages of voids (p > 0.05) at each level independent from shaping systems. Continuous wave technique had significantly higher gutta-percha percentages and lower sealer percentages than lateral condensation technique on average in all sections(p<0.05). However, there were no statistically significant differences in the percentages of gutta-percha, sealer and voids between *Reciproc Blue* and H*yflex EDM* systems at any level (*p>0*.*05*). Also, for the percentages of gutta-percha, sealer and voids, the difference between the level of sections (2mm, 4mm, 6mm and total) was not significant irrespective of filling techniques (*p* >0.05). Tables 5, 6 and 7 show all descriptive statistics for the variables and *p* values for all group differences in C2-type.

**Table 5:** Mann-Whitney U and Kruskal-Wallis tests results with descriptive statistics of C2-type’s median percentage values of gutta-percha, sealer and voids according to filling techniques, shaping system and level of sections.

		Gutta-Percha			Sealer			Voids		
	*n*	*Median*	*IQR*	*p*	*Median*	*IQR*	*p*	*Median*	*IQR*	*p*
*Filling Techniques*										
LC	30	80.73^a^	10.03	< .001	18.70^a^	9.81	< .001	0.00	0.05	.32
CW	30	97.30^a^	5.06		2.21^a^	4.99		0.00	0.39	
*Shaping System*										
R	30	90.78	15.24	.90	9.2	15.60	.88	0.00	0.32	.77
H	30	88.06	20.30		11.97	19.55		0.00	0.07	
*Level of Sections*										
2mm	20	87.88	16.97	.16	12.15	17.10	.16	0.00	0.49	.052
4mm	20	93.47	11.42		6.52	11.72		0.00	0.00	
6mm	20	88.54	21.78		11.21	21.26		0.00	0.29	
Total	20	89.64	16.53		10.3	16.50		0,18	0.38	

^a^ Statistically significant difference between groups at level of p < .001

**Table 6:** Descriptive statistics and Kruskal-Wallis test results on the percentages of gutta-percha, sealer and voids according to level of sections in C2-type.

		Gutta-Percha			Sealer			Voids		
	*n*	*Median*	*IQR*	*p*	*Median*	*IQR*	*p*	*Median*	*IQR*	*p*
*2mm*										
R-LC	5	78.33^a^	9.43	< .01	20.54^a^	9.43	< .01	0.00	0.56	.29
H-LC	5	76.67^a^	7.39		23.32^a^	7.40		0.00	0.00	
R-CW	5	93.43^a^	5.75		6.02^a^	6.25		0.37	0.57	
H-CW	5	94.09^a^	9.53		5.90^a^	8.94		0.00	1.58	
*4mm*										
R-LC	5	86.21^a^	8.42	< .01	13.78^a^	8.69	< .01	0.00	0.26	.25
H-LC	5	87.25^a^	3.17		12.74^a^	3.17		0.00	0.00	
R-CW	5	97.91^a^	2.16		1.71^a^	1.75		0.00	0.59	
H-CW	5	96.91^a^	7.77		3.08^a^	7.76		0.00	0.00	
*6mm*										
R-LC	5	75.06^a^	10.70	< .01	24.93^a^	10.83	< .01	0.00	0.13	.07
H-LC	5	76.94^a^	5.63		22.74^a^	5.52		0.81	0.95	
R-CW	5	96.95^a^	4.12		3.04^a^	4.16		0.00	0.27	
H-CW	5	93.33^a^	2.16		1.11^a^	2.17		0.00	0.29	
*Total*										
R-LC	5	81.05^a^	7.98	< .01	18.94^a^	7.98	< .01	0.12	0.24	.70
H-LC	5	80.23^a^	4.00		19.26^a^	5.57		0.40	0.45	
R-CW	5	96.38^a^	1.98		3.59^a^	2.05		0.23	0.17	
H-CW	5	97.16^a^	4.19		2.26^a^	3.95		0.01	0.53	

^a^ Statistically significant difference between groups at level of p < .01

**Table 7:** Descriptive statistics and Kruskal-Wallis test results on the percentages of gutta-percha, sealer and voids according to level of sections independent from shaping system in C2-type.

		Gutta-Percha			Sealer			Voids		
	*n*	*Median*	*IQR*	*p*	*Median*	*IQR*	*p*	*Median*	*IQR*	*p*
*2mm*										
LC	10	77.04^a^	9.59	< .01	22.96^a^	9.59	< .01	0.00	0.00	.07
CW	10	93.76^a^	6.90		5.96^a^	6.84		0.19	0.77	
*4mm*										
LC	10	86.73^a^	3.03	< .01	13.26 ^a^	3.10	< .01	0.00	0.00	.54
CW	10	97.87^a^	2.98		1.90 ^a^	2.97		0.00	0.11	
*6mm*										
LC	10	76.59^b^	6.06	< .001	23.00 ^b^	6.35	< .001	0.09	0.86	.41
CW	10	98.05^b^	2.32		1.92 ^b^	2.42		0.00	0.26	
*Total*										
LC	10	80.64^b^	5.20	< .001	19.10 ^b^	6.55	< .001	0.15	0.42	.94
CW	10	96.77^b^	2.77		2.99 ^b^	2.55		0.21	0.32	

^a^ significant difference between groups at level of p < .01,

^b^ p < .001

**Fig 8:**
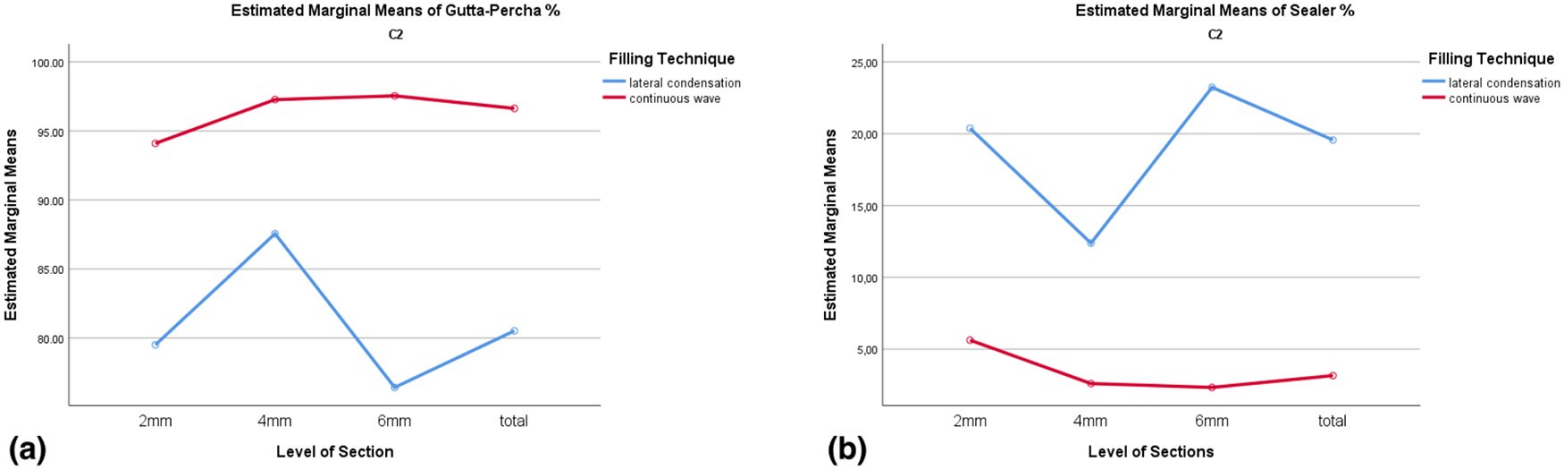
Interaction effects of filling techniques and level of sections on (A)gutta-percha (B)sealer percentages in C2-type.

Due to lack of statistical significance in changes in the percentages of gutta-percha, sealer and voids depending on the level of sections (2 mm, 4 mm, 6 mm, 8 mm and total) (*p* > 0.05), a test to explore interaction effects was not conducted (Fig 8A and 8B).

**Fig 9:**
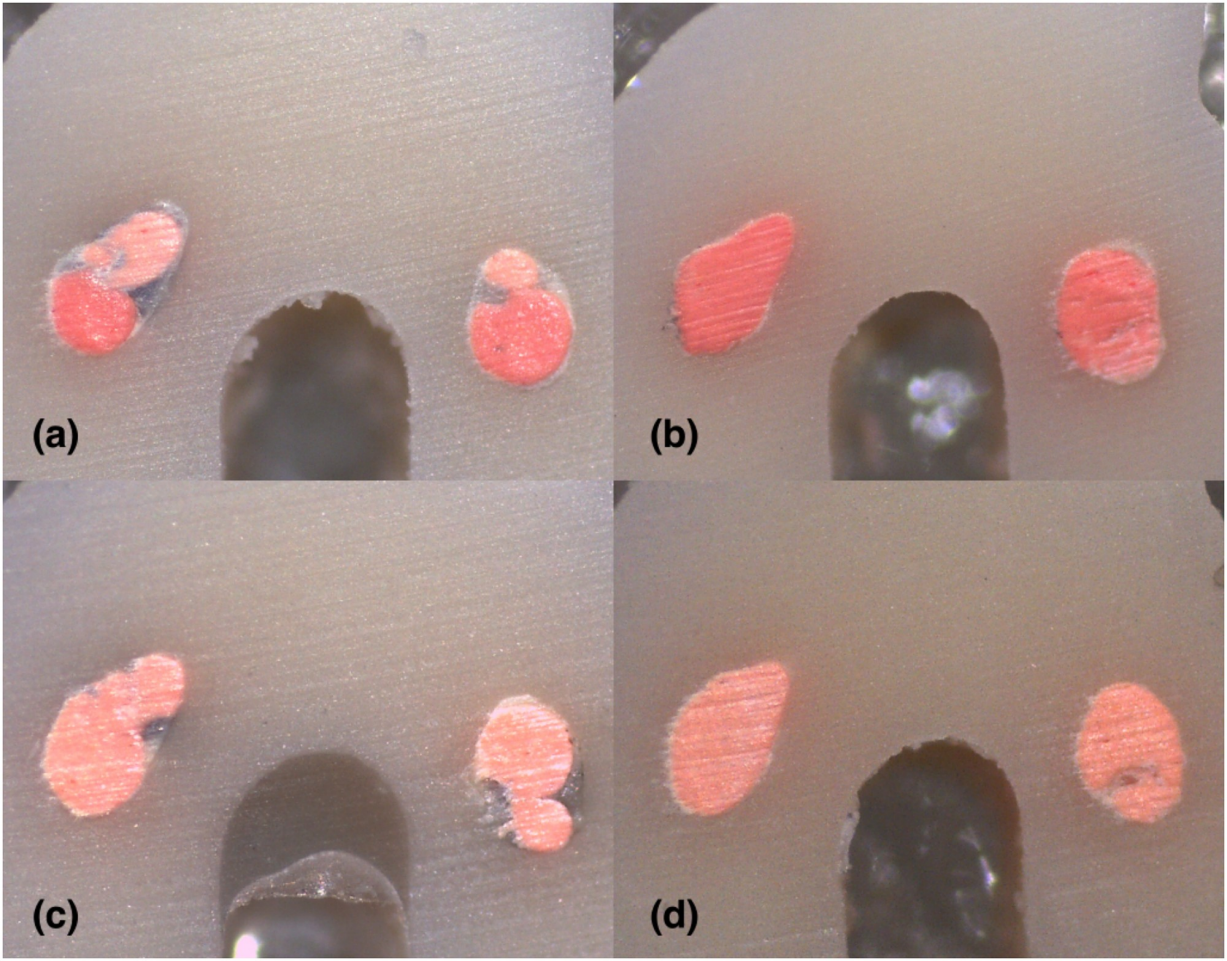
Stereomicroscope images of 2 mm sections instrumented with Hyflex EDM: (A) LC (B) CW; Stereomicroscope images of 2 mm sections instrumented with Reciproc Blue: (C) LC (D) CW in C2-type.

**Fig 10:**
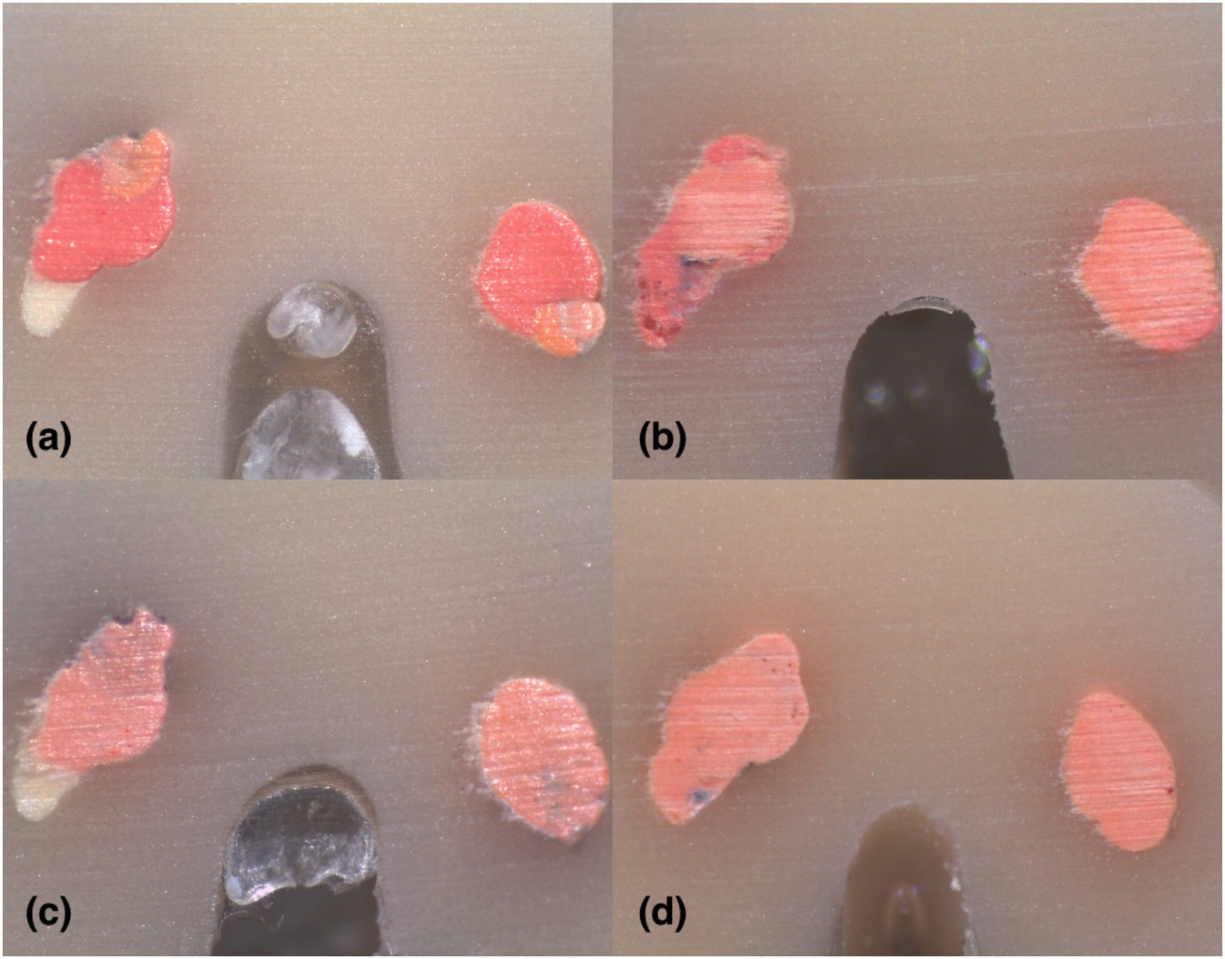
Stereomicroscope images of 4 mm sections instrumented with Hyflex EDM: (A) LC (B) CW; Stereomicroscope images of 4 mm sections instrumented with Reciproc Blue: (C) LC (D) CW in C2-type.

**Fig 11:**
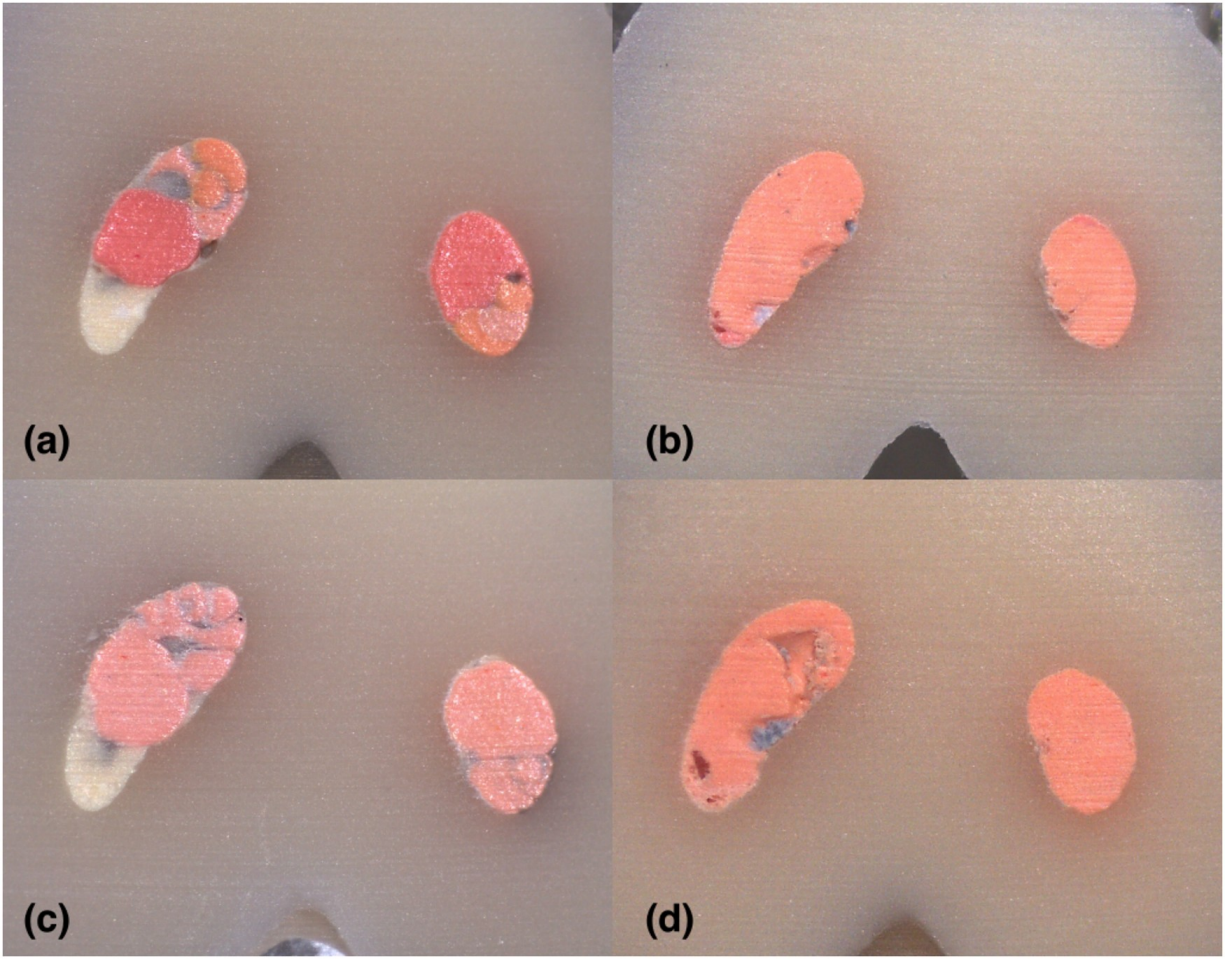
Stereomicroscope images of 6 mm sections instrumented with Hyflex EDM: (A) LC (B) CW; Stereomicroscope images of 6 mm sections instrumented with Reciproc Blue: (C) LC (D) CW in C2-type.

For the investigation of both shaping systems’ and filling techniques’ impact on the percentages of gutta-percha, sealer and voids by controlling the sectional levels, non-parametric Kruskal-Wallis test was conducted. The analysis showed a statistically significant difference in percentages of gutta-percha and sealer at all levels of sections (p<0.05) and no statistically significant difference in the percentages of voids at any level of sections between the filling techniques (p>0.05). CW group had more gutta-percha and less sealer versus LC group in all sections (2, 4, 6 mm) and total area (p<0.05) (Figs 9–11). In 6-mm sections CW group had the highest percentages of gutta-percha and lowest of sealer. LC group had the lowest gutta-percha and highest sealer percentages in 6 mm sections (p<0.05) (Fig 11).

## Discussion

The null hypothesis was rejected since the CW technique was associated with higher percentages of gutta-percha compared to LC technique in total areas of C1 and C2-types and instrumentation techniques did not alter obturation quality.

Conversely, CW had the least percentages of gutta-percha at 2-mm section of C1-type compared to LC, which was in part similar to the results of study by Gok et al. [33]. On the other hand, contrary to their findings, CW was found to be superior to LC at 4 and 6-mm sections. This could be explained by the use of rotary or reciprocating systems instead of hand files in the isthmus area of C1-type which aid flow of thermaplasticized gutta-percha by creating a favorable space in present study. Although 3–4 gutta-percha cones were used to perform the CW technique in C1-type to establish the stability of cones in high volume and taper of canal at apical during down-pack procedure, an excessive amount of sealer and low gutta-percha mass found at 2-mm sections could be attributed to insufficient heat transfer to gutta-percha independently from clinical experience of operator [36]. Therefore, a modified CW technique with the addition of laterally compacted 0.2 taper gutta-percha cones at the apical area should be preferred for precision with this particular technique at any level of C1-type canal area. Also, multiple burst of heats from multiple points could be applied during down-pack procedure to facilitate softening of gutta-percha at the apical level.

Although one of the teeth chosed to be replicated with 3D printing had C2 and C3 root canal morphology at different sections, it was referred as C2-type based on the orifice appearance for the ease of labeling. This shows the difficulty of exact classification of C-shaped canals due to high variability of shapes throughout the root length [18].

In C2-type, lower gutta-percha percantages at 6-mm sections filled with LC technique could be attributed to pronounced comma like C2-shape at this level differently from other levels which have C3–type morphologies resembling an irregular shaped oval canal. On the contrary, CW technique was found to be superior at each level in terms of obturation quality compared to LC in C2-type. Consistent with the results of this study, Gencoglu et al.[37] also reported that thermoplasticized gutta-percha filling techniques were superior to laterally condensed gutta-percha in regard to higher gutta-percha content.

In this study, mean gutta-percha percentages, irrespective of filling techniques, at 2-mm and 4-mm C3-shaped levels of C2-type resin replica were 86.80% and 92.41% respectively and 86.98% at 6 mm C2-shaped level of C2-type. For C1-type resin replica, the corresponding values were 75.64%, 89.62% and 90.85% for 2, 4 and 6 mm, respectively. Ordinola-Zapata et al. [31] reported mean percentages of 74.5%, 93.32% and 97.92% and Soo et al [24] reported 91.75%, 97.57% and 98.26% from apical to coronal sections. As this is the first study that used a standardized 3D printed resin model containing both C2 and C3 morphology at different sections (Figs 9–11), it was not conceivable to compare our results directly with previous studies which used resin simulated C1 morphology with a C4 apical part [24] or extracted human teeth with various types of C morphologies based on radiological classification which prevents the standardization of samples [31]. Also, use of mostly thermoplasticizing techniques in these studies might have resulted in higher mean percantages of gutta-percha due to the success of thermoplasticized techniques in filling irregular shaped canals compared to design of present study that used only cold lateral condensation technique as control which could have lowered the mean values.

In the present study, Reciproc Blue and Hyflex EDM shaping systems were not significantly different on the filling quality of C-shaped root canals. Resin simulated replicas that were obtained from extracted human teeth were instrumented and then replicated for filling in order to mimic instrumentation system shapes. However, in studies of Gok et al. [33] and Soo et al. [24] extracted human teeth was first instrumented with one multiple file rotary system or hand filing and then replicated. Depending on the differences in the hardness value of resin 3D printing material from dentin, there may be slight differences compared to human teeth [38] which could be considered as a limitation of this study. Additionally, since instrumentation with each system was performed only once, it may not reflect the possible hand movements and force appliance of the operator. In this study, remaining resin thickness of the groove area at 4-mm level of C1-type which was instrumented with Reciproc Blue was found to be less than 0.3 mm in contrast to Hyflex EDM instrumented group which stayed in safe range values suggested by Lim et al.[39] to stand out against compaction forces during filling without the risk of perforation (Fig 5). Also, another concern is excessive pressure applied during lateral compaction of gutta-percha could create micro-cracks[40] in extensively flared dentinal canal wall of human teeth which will eventually increase the risk of vertical root fracture as well as pressure effect of hydraulic forces created during continuous wave obturation[41, 42] and forces applied with pluggers could cause to the same result. Thus, the shaping characteristics of Reciproc Blue and Hyflex EDM systems and the impact on dentin thickness values and vertical root fracture rates on different C-shaped canals need to be separately investigated in further studies.

In this study, the mean time spent for filling by LC technique in C1-type was found to be shorter compared to results of Soo et al.[24] and longer compared to results of Gok et al. [33]. This outcome could be attributed to uniqueness of all teeth morphology. The mean time spent for filling by CW technique in C1 type was longer than that reported by Soo et al. and Gok et al. Differences in the mean time required with CW technique could be related to differences in canal volumes between studies and obturation devices. The mean time required for obturation of C1 type was longer than C2 and obturation time of LC was longer versus CW in the present study. This could be explained by higher canal volume of C1-type compared to C2-type.

Even though LC is widely used and the most accepted obturation method, the mean time spent for LC technique, used for treatment of a second molar of a patient with restricted mouth opening, in a real life scenario would be longer than that observed in in-vitro studies. This is because most orifices of C-shapes are 3 mm below the cemento-enamel junction [18] which may led to improper penetration of finger spreader and accessory cones.

Standardization of samples with respect to canal morphology, configuration, taper and apical diameter for the comparison of different filling techniques in differently instrumented canals has a major impact on the credibility of results. In that regard, 3D printing technology used in this study has a great importance and could also have a remarkable role in pre-clinical teaching of dental students by providing standardized different root canal anatomies selected and reconstructed by tutors which will pave the path to a better clinical internship success.

## Conclusion

Within the limitations of this study, continuous wave obturation was more effective than lateral condensation technique on both of C1 and C2-types, except for the apical 2mm section of C1-type, suggesting the need for a modified CW technique. 3D printing technology could be the future of standardized in vitro studies and teaching methods in endodontics.
